# Global Trends and Attributable Risk Factors in the Disease Burden of Lower Respiratory Infections

**DOI:** 10.3390/tropicalmed10070180

**Published:** 2025-06-26

**Authors:** E Yu, Chunhui Li

**Affiliations:** Institute of Data Science and Big Data Technology, School of Mathematics and Physics, Wuhan Institute of Technology, Wuhan 430205, China; 22310010026@stu.wit.edu.cn

**Keywords:** lower respiratory infections, worldwide disease burden, variation trend, age–period–cohort model, risk factors

## Abstract

Background: Lower respiratory infections (LRIs) are the leading cause of the global disease burden, accounting for millions of deaths each year. Methods: Data on LRIs, including deaths, disability-adjusted life years (DALYs), and incidence, were obtained from the Global Burden of Disease Study 2021. Joinpoint regression was employed to assess temporal trends in the LRIs’ burden, while the age–period–cohort model was used to evaluate age, period, and cohort effects. Pearson’s correlation coefficients were calculated to examine the relationship between DALYs attributable to risk factors and the socio-demographic index (SDI). Results: Over recent decades, the average annual percentage change in age-standardized mortality rate, age-standardized DALYs rate, and age-standardized incidence rate of LRIs globally were −2.4%, −3.5%, and −1.3%, respectively. Notably, the LRIs’ burden dropped considerably from 2019 to 2021. The disease burden was higher among children under five and individuals over 60 compared to other age groups. In terms of gender, males had a higher burden. The age-standardized DALYs rate of LRIs was strongly and negatively correlated with SDI (*r* = −0.84; *p* < 0.05). *Streptococcus pneumoniae* remained the leading pathogen, followed by *Staphylococcus aureus*, and *Klebsiella pneumoniae*. Conclusions: In recent years, the global burden of LRIs has declined, but regional, gender, and age disparities persist. Targeted measures are needed to address high-risk populations and major risk factors.

## 1. Introduction

Lower respiratory infections (LRIs), mainly caused by bacteria (such as *Streptococcus pneumoniae*, *Haemophilus influenzae type b*, *Klebsiella pneumoniae*, and *Staphylococcus aureus*) and viruses (including influenza and respiratory syncytial virus (RSV)), are the leading cause of global mortality and morbidity, with severe complications like pleural effusion, heart failure, and anemia [1,2]. Although the global burden of LRIs has declined in recent decades due to improved healthcare, vaccination, and public health initiatives, they remain a major health challenge. According to the Global Burden of Disease Study 2021 (GBD 2021), LRIs caused approximately 2.2 million deaths, ranking them as the seventh leading cause of death worldwide [3]. Notably, *Streptococcus pneumoniae* remains the top bacterial cause of LRI-related deaths (505,000 annually), followed by *Staphylococcus aureus* (424,000), and *Klebsiella pneumoniae* (176,000) [4]. Meanwhile, the RSV continues to be a major viral contributor, responsible for 33 million LRIs’ cases and 3.6 million hospitalizations in children under five each year [5].

Regional disparities persist, with some areas experiencing disproportionately high LRI burdens driven by inequities in healthcare access and infrastructure [6]. However, existing studies have primarily focused on the burden of LRIs in specific countries or regions, while fewer analyses have been conducted on a global scale [7,8]. Most studies focus on children under five and the elderly, as they bear a severe disease burden [1,9]. However, the disease burden among older children and adults has often been overlooked in research [10,11,12].

LRIs are influenced by various factors, including environmental contamination, nutrition, and infrastructure, especially in low- and middle-income countries [7,13,14]. Despite the widely acknowledged multifactorial nature of LRIs, many studies have narrowly focused on individual risk factors or neglected to consider the collective impact of these factors on LRIs [13,15,16,17]. The limited scope reduces the applicability of findings in designing comprehensive interventions and policies that address the root causes of LRIs.

Global analyses of the LRIs’ burden are limited, with existing studies like GBD 2019 requiring updates to reflect recent trends. This study aims to provide an in-depth, up-to-date analysis of LRIs, centering on mortality, disability-adjusted life years (DALYs), and incidence. Additionally, it will explore age- and sex-specific patterns, identify disparities, and examine the impact of risk factors and the socio-demographic index (SDI), including etiological analysis.

## 2. Materials and Methods

### 2.1. Data Sources

The data were obtained from GBD 2021. Specific information related to LRIs, including statistics on deaths, DALYs, incidence, age-standardized mortality rate (ASMR), age-standardized disability-adjusted life years rate (ASDR), and age-standardized incidence rate (ASIR), were extracted. Additionally, the SDI for 204 countries and territories was collected. To further analyze the burden of LRIs, the study examined multiple attributable risk factors, including smoking, secondhand smoke, household air pollution from solid fuels, child underweight, ambient particulate matter pollution, non-exclusive breastfeeding, child wasting, child stunting, short gestation, low birth weight, high temperature, low temperature, and no access to handwashing facilities, while concurrently analyzing 18 etiological agents of LRIs: *Streptococcus pneumoniae*, *Staphylococcus aureus*, *Klebsiella pneumoniae*, *Pseudomonas aeruginosa*, *Escherichia coli*, the influenza virus, *Acinetobacter baumannii*, *Legionella* spp, *Mycoplasma pneumoniae*, *Haemophilus influenzae*, group B Streptococcus, *Chlamydia* spp, fungi, RSV, *Enterobacter* spp, polymicrobial infections, other bacterial/viral pathogens, and other viral etiologies of LRIs.

The GBD 2021 study is a systematic analysis providing comprehensive estimates of incidence, prevalence, mortality, and DALYs by sex, age, and subnational location across 204 countries and territories from 1990 to 2021. The GBD integrates diverse data sources—including vital registration, verbal autopsy, census and sample surveys, surveillance, cancer registry, police records, open-source databases, and minimally invasive tissue sampling—to ensure robust and representative global health assessments. In low SDI countries, where complete vital registration systems are often lacking, the GBD compensates by integrating alternative data sources, such as verbal autopsy and various surveys. To address incomplete and biased data, GBD applies a range of data cleaning procedures, including the redistribution of garbage codes, exclusion of low-quality data, and identification and manual review of outliers. Additionally, the GBD employs the Cause of Death Ensemble Modeling (CODEm) framework to mitigate the impact of data quality variation on estimates. In specific countries, such as India, missing data on sex, age, and location in the Sample Registration System and Medical Certification of Causes of Death are addressed using a multidimensional log-linear model, ensuring data completeness and accuracy of estimates [18]. Relevant data can be accessed through the GBD Data Tool (https://vizhub.healthdata.org/gbd-results/ (accessed on 26 August 2024)). The SDI is a composite measure of lag-distributed income per capita, average years of education, and fertility rates among females younger than 25 years, with values ranging from 0 to 1. Based on the SDI level, the GBD study categorizes the world into five distinct groups [19].

### 2.2. Statistical Analysis

This study analyzes the global burden of LRIs, including mortality, DALYs, and incidence, across 204 countries, 21 regions, and five SDI categories. To analyze temporal trends, Joinpoint regression was used to calculate the average annual percentage change (AAPC) in ASMR, ASDR, and ASIR, with 95% confidence intervals (CI) stratified by sex and age group. Additionally, the age-specific burden was further explored through stratified analyses. The age–period–cohort (APC) model was employed to systematically analyze the effects of age, period, and cohort on mortality, DALYs, and incidence in LRIs (https://analysistools.cancer.gov/apc/ (accessed on 27 August 2024)) [20]. Ages were categorized into 20 five-year groups, ranging from 0 to >95 years. For mortality, the periods were divided into nine five-year intervals from 1980 to 2024. Similarly, the periods for DALYs and incidence were divided into seven five-year intervals from 1990 to 2024. The birth cohorts were categorized into 28, 26, and 26 five-year groups for mortality, DALYs, and incidence, respectively.

The study also examined the percentage of LRIs DALYs attributable to various risk factors in 2021, stratified by age and sex. The AAPC of ASDR attributable to risk factors was calculated from 1990 to 2021, with analyses by demographic groups. Additionally, Pearson’s correlation coefficient between ASDR and SDI in 2021 was calculated to explore their relationship.

Data processing and visualization were conducted using R (version 4.1.0). A *p*-value of less than 0.05 was considered statistically significant.

## 3. Results

### 3.1. Distribution and Trends in LRIs’ Burden

**Figure 1:**
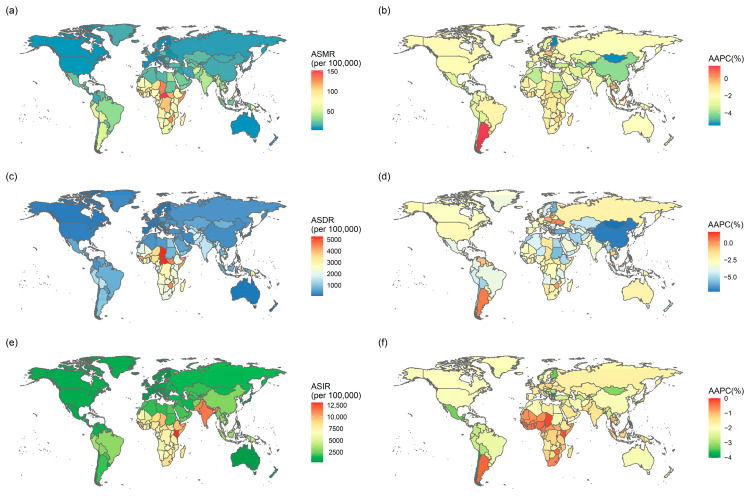
Global burden and temporal trends of lower respiratory infections in countries and territories. (**a**) ASMR in 2021; (**b**) AAPC of ASMR from 1980 to 2021; (**c**) ASDR in 2021; (**d**) AAPC of ASDR from 1990 to 2021; (**e**) ASIR in 2021; (**f**) AAPC of ASIR from 1990 to 2021. ASMR, age-standardized mortality rate; AAPC, average annual percentage change; ASDR, age-standardized disability-adjusted life years rate; ASIR, age-standardized incidence rate.

In 2021, LRIs caused approximately 2.2 (95% uncertainty interval (UI): 2.0 to 2.4) million deaths worldwide, with a global ASMR of 28.7 (95% UI: 26.0 to 31.1) per 100,000. The AAPC was −2.4% (95% CI: −2.6 to −2.3%; *p* < 0.05) from 1980 to 2021. Regionally, Central sub-Saharan Africa recorded the greatest ASMR, at 106.7 (95% UI: 81.8 to 138.6) per 100,000 in 2021 (Supplementary Table S1). The country with the highest ASMR in 2021 was the Central African Republic, followed by Eritrea and Chad (Figure 1a). Between 1980 and 2021, ASMR showed a decline in most countries, with the largest reductions observed in Finland, Mongolia, and Kyrgyzstan. Only a few countries, such as Argentina, exhibited an increasing trend (Figure 1b).

In 2021, the number of DALYs due to LRIs worldwide was estimated at 82.5 (95% UI: 72.6 to 93.4) million, with an ASDR of 1168.8 (95% UI: 1017.0 to 1336.9) per 100,000. The AAPC was −3.5% (95% CI: −3.6 to −3.4%; *p* < 0.05) from 1990 to 2021. The greatest ASDR of LRIs was concentrated in Western sub-Saharan Africa, at 3258.7 (95% UI: 2537.0 to 4045.2) per 100,000 (Supplementary Table S1). Similarly, the three countries with the highest ASDR were Central African Republic, Chad, and Lesotho (Figure 1c). The majority of countries demonstrated a downward trend in ASDR from 1990 to 2021. Mongolia, China, and Albania showed a considerable decline, while Niue displayed an upward trend (Figure 1d).

LRIs accounted for 343.6 (95% UI: 325.2 to 363.5) million incidence cases in 2021, with an ASIR of 4283.6 (95% UI: 4057 to 4524.9) per 100,000. The AAPC was −1.3% (95% CI: −1.4 to −1.2%; *p* < 0.05) since 1990. Regionally, the ASIR of LRIs was the greatest in South Asia, reaching 11,101.2 (95% UI: 10,358 to 11,891.2) per 100,000 in 2021 (Supplementary Table S1). Kenya recorded the highest ASMR, followed by Nepal and India (Figure 1e). From 1990 to 2021, the ASIR of LRIs exhibited a notable decline in Albania, Bulgaria, and Chile (Figure 1f).

Between 1980 and 2019, the global number of deaths from LRIs declined by 25.53% (95% UI: 35.70% to 13.77%). From 2019 to 2021, there was a great decrease of 14.4% (95% UI: 14.5% to 14.2%). The DALYs for LRIs decreased by 49.39% (95% UI: 49.29% to 49.32%) from 1990 to 2019, and by 20.12% (95% UI: 20.75% to 19.52%) from 2019 to 2021. The number of incident cases increased by 17.62% (95% UI: 17.16 to 18.73) from 1990 to 2019 but decreased by 6.93% (95% UI: 6.85 to 6.91) from 2019 to 2021 (Supplementary Table S2, Figure S1). Prior to 2019, the number of deaths and DALYs due to LRIs showed a decreasing trend in the low, low-middle, middle, and high-middle SDI regions, while the incidence increased across all five SDI regions. From 2019 to 2021, all five SDI regions experienced declines in deaths, DALYs, and incidence (Supplementary Table S2, Figure S2).

### 3.2. Gender and Age Patterns of LRIs’ Burden

In 2021, the ASMR, ASDR, and ASIR of LRIs were globally higher in males (Supplementary Figure S3a). The ASMR for both males and females demonstrated a downward trajectory from 1980 to 2021. Similar declines were observed in ASDR and ASIR from 1990 to 2021, with females showing a more pronounced reduction across all three indicators (Supplementary Figure S3b).

**Figure 2:**
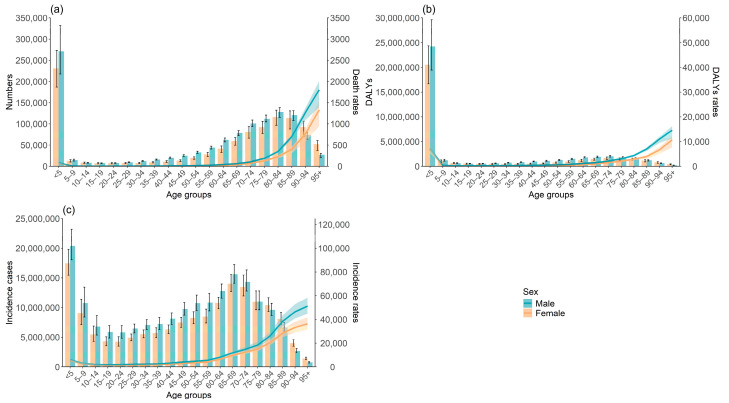
Global burden of lower respiratory infections by age and sex in 2021. (**a**) Deaths; (**b**) DALYs; (**c**) incidence. DALYs, disability-adjusted life years.

In 2021, global LRIs’ mortality was slightly elevated in males, peaking in those aged 95 and older. The greatest number of deaths occurred in the <5 years age group, followed by a decline with age, before peaking again in the 80–84 age group (Figure 2a). The global LRIs DALYs’ rate was also higher in males, with the highest rates observed in the <5 years age group and then decreasing with age until the 15–19 years age group, before rising slowly. Similarly, the number of DALYs peaked in the <5 years age group (Figure 2b). The global LRIs incidence rate showed a declining trend up to the 25–29 years age group, after which it escalated with age. The highest number of incidence cases occurred in the 65–69 and 5–9 years age groups (Figure 2c).

### 3.3. The Effect of Age, Period, and Cohort on LRIs Burden

**Figure 3:**
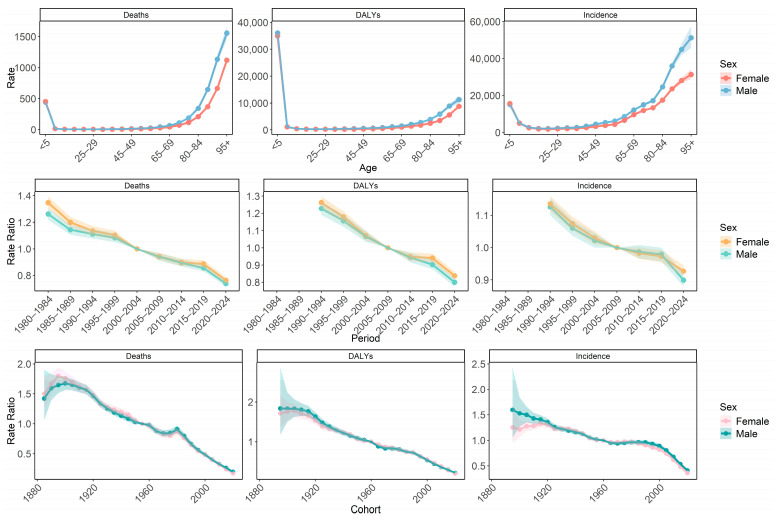
Age–period–cohort model analysis of deaths (1980–2021), d DALYs (1990–2021) and incidence (1990–2021) rates of lower respiratory infections globally by sex. DALYs, disability-adjusted life years.

The study revealed that the risk of mortality due to LRIs decreased and then increased with age for both sexes. The highest mortality risk was observed in the 95 years and older age group, followed by the <5 years age group. After the 75–79 age group, males had a greater risk of mortality than females. The mortality risk declined in both sexes over time. In the period preceding 2000–2004, the risk of mortality was marginally higher for females than for males. The mortality risk across birth cohorts fluctuated, initially rising, then falling, with a temporary spike in 1980 (Figure 3).

Globally, the age effect analysis showed that the risk of DALYs for LRIs initially decreased and then increased with age, with the highest risk observed in the <5 years age group. Both the period and cohort effects exhibited a downward trend in both males and females (Figure 3).

The risk of morbidity displayed a pattern of decline followed by an escalation with age, peaking in the 95 years and older age group. After the 65–69 age group, males exhibited a greater morbidity risk than females. The period effects demonstrated a decreasing trend in both sexes. The cohort effects also showed a declining trend, with females displaying a higher morbidity risk than males before 1920 (Figure 3).

### 3.4. DALYs of LRIs Attributable to Risk Factors

**Figure 4:**
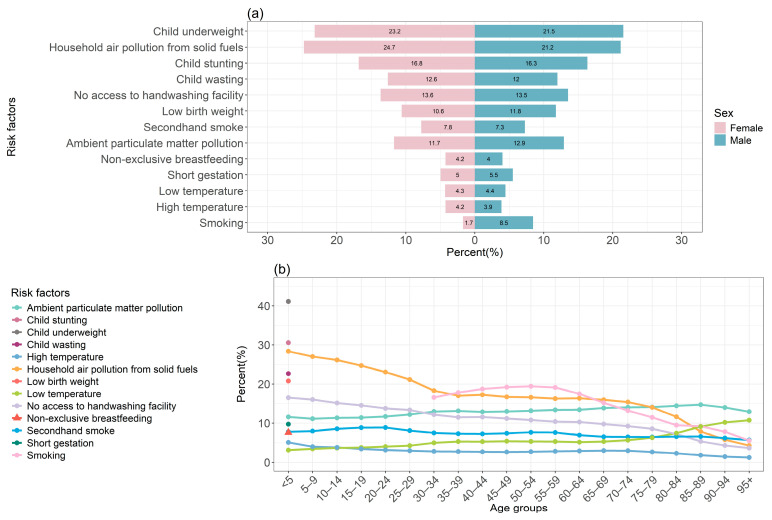
Percentage of DALYs due to lower respiratory infections attributable to risk factors by sex and age globally in 2021. (**a**) Sex; (**b**) age. DALYs, disability-adjusted life years.

In 2021, the highest percentage of DALYs for LRIs in males was attributed to child underweight, while in females, it was due to household air pollution from solid fuels. Additionally, the percentage of DALYs related to smoking differed by sex (Figure 4a). Child underweight was the primary risk factor for the percentage of DALYs due to LRIs in children under five. Household air pollution from solid fuels was the main risk factor for ages 5–34, while smoking was the leading risk factor for ages 35–64. For individuals aged 80 and above, ambient particulate matter pollution was the primary risk factor for DALYs due to LRIs (Figure 4b).

**Figure 5:**
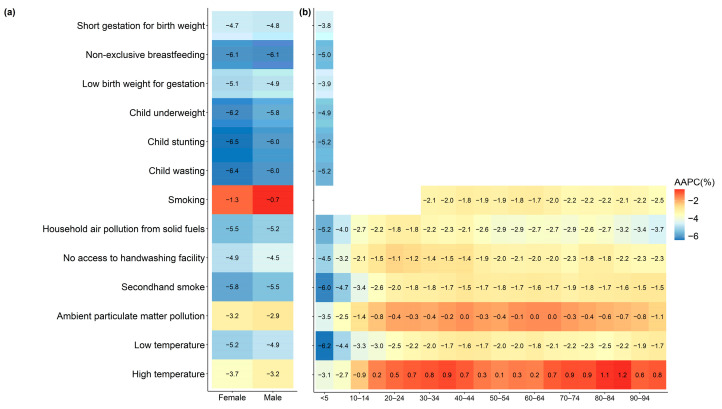
AAPC of lower respiratory infections DALYs attributable to risk factors by sex and age globally from 1990 to 2021. (**a**) Sex; (**b**) age. AAPC, average annual percentage change; DALYs, disability-adjusted life years.

The study calculated the AAPC by gender and age group to analyze trends in LRIs DALYs. From 1990 to 2021, the DALYs rates of LRIs attributable to all risk factors showed a declining trend in both males and females. Among the factors, the AAPC of DALYs rates attributable to smoking was the largest, indicating the slowest decline. The reduction in DALY rates was more pronounced in the under-10 age group, while most risk factors exhibited a smaller decrease in middle-aged and older groups. DALYs’ rates of LRIs due to high temperature followed an increasing trend in age groups over 15 years (Figure 5).

A significant negative relationship was found between the ASDR of LRIs and the SDI level (*r* = −0.84; *p* < 0.05). Of these factors, household air pollution from solid fuels (*r* = −0.83; *p* < 0.05) and lack of access to handwashing facilities (*r* = −0.81; *p* < 0.05) displayed a strong negative correlation with the SDI. Smoking (*r* = −0.46; *p* < 0.05) and high temperatures (*r* = −0.49; *p* < 0.05) showed weaker negative correlations (Supplementary Figure S4). The relationship between the ASDR of LRIs attributable to other risk factors and SDI is shown in Supplementary Figure S4.

### 3.5. Etiologies of LRI

**Figure 6:**
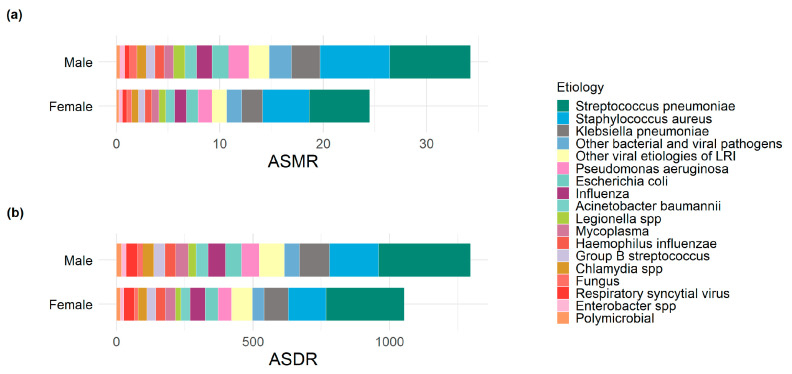
Etiology distribution of global lower respiratory infections ASMR and ASDR by sex in 2021. (**a**) ASMR; (**b**) ASDR. ASMR, age-standardized mortality rate; ASDR, age-standardized disability-adjusted life years rate.

In 2021, the leading pathogen contributing to global LRIs’ ASMR and ASDR was *Streptococcus pneumonia* [ASMR: 6.69 (95% UI: 5.98 to 7.35), ASDR: 309.90 (95% UI: 264.44 to 357.22)], followed by *Staphylococcus aureus* [ASMR: 5.43 (95% UI: 4.89 to 5.90), ASDR: 156.80 (139.44,176.08)] and *Klebsiella pneumonia* [ASMR: 2.32 (95% UI: 2.09 to 2.58), ASDR: 99.46 (95% UI:84.65 to 115.82)], with a higher burden observed in males than in females (Figure 6, Supplementary Table S3). From 1990 to 2021, all etiologies of LRIs demonstrated declines in ASMR and ASDR. The most pronounced reduction was observed for RSV-associated infections [ASMR (AAPC: −5.38, 95% CI: −5.84 to −4.92, *p* < 0.05), ASDR (AAPC: −5.40, 95% CI: −5.82 to −4.97, *p* < 0.05)] (Supplementary Table S3).

## 4. Discussion

The study provides a comprehensive analysis of the global burden of LRIs in recent decades, revealing pronounced regional differences in the disease burden. The findings indicate a considerable decline in the global ASMR, ASDR, and ASIR for LRIs, consistent with previous studies [14]. The reduction may be due to improvements in public health programs and practices that have been implemented since the late 19th century, as well as advancements in tools for the prevention, diagnosis, and treatment of infectious diseases [21]. Accurate identification of pathogens in LRIs facilitates targeted therapy, thereby improving treatment efficacy and accelerating patient recovery [22]. Pathogen detection techniques for LRIs are shifting from inefficient traditional culture-based methods to high-sensitivity, broad-spectrum, and rapid approaches based on molecular technologies, exemplified by metagenomic next-generation sequencing, providing strong support for accurate diagnosis and targeted therapy [22,23,24].

Between 2019 and 2021, we observed marked declines in the global burden of LRIs. This decline may be related to non-pharmaceutical interventions implemented during the COVID-19 pandemic. A study by Bender et al. [25] suggests that pandemic-related measures—including stay-at-home orders, school/community closures, and mask mandates—effectively reduced the incidence of respiratory infections (including LRIs) by suppressing COVID-19 and other respiratory viruses in 2020−2021. Several studies have also demonstrated that COVID-19 mitigation strategies effectively reduced the transmission of common epidemic respiratory viruses [26,27]. Vaccination is a key strategy for preventing LRIs, particularly for pathogens including influenza virus, RSV, and *Streptococcus pneumoniae* [28]. From 1990 to 2019, substantial declines in LRIs mortality from *Haemophilus influenzae* and *Streptococcus pneumoniae* were primarily attributable to vaccination programs [25].

Our results indicated that although ASMR, ASDR, and ASIR of LRIs have decreased in recent decades, the numbers of incidence cases of LRIs in 2021 were higher than in 1990. Certain regions, such as sub-Saharan Africa, continue to experience disproportionately higher disease burdens. These disparities are likely due to environmental risk factors and systemic barriers, including limited access to diagnostic tools, specialist care, and essential treatments [6]. Furthermore, socio-economic status significantly impacts disease burden. Lower socio-economic status, combined with weak social protections, correlates strongly with higher rates of infectious diseases like LRIs [29]. These findings highlight the need for targeted public health interventions. Despite the progress in reducing the global impact of LRIs, further focused efforts are needed, particularly in low-income and resource-limited areas.

The study has found that the burden of LRIs was generally higher in males than females. Some studies have indicated that males tend to develop respiratory tract infections more frequently than females [30,31]. Multiple factors drive the disparity, such as sex-based immune differences. Males generally mount weaker immune responses than females, raising their respiratory infection risk [30]. Additionally, lifestyle factors such as smoking, which are more common in men, may increase their vulnerability to LRIs. Notably, females tend to possess more robust immune systems than males [32]. These immune differences, together with behavioral and environmental factors, are likely contributing to the observed gender disparities in LRIs burden.

In 2021, LRIs mortality was the highest among children under five, reflecting their increased vulnerability due to immune system activation and cytokine release [33]. Early diagnosis, testing, and vaccine development are critical to reducing incidence and mortality in the group. The rising mortality risk with age, particularly in individuals over 60, highlights the need for effective LRIs management in older adults, as their outcomes are exacerbated by comorbidities and frailty. LRIs prevalence in the elderly is influenced by factors such as previous hospitalizations, antimicrobial treatments, and antibiotic resistance [34]. Although the burden is the heaviest among young children and older adults, the incidence remains high among older children and adults. Therefore, the LRIs burden in these populations should also be acknowledged, and effective measures should be implemented to reduce their incidence.

Age effects indicated that the risk of mortality, DALYs, and incidence initially declined and then increased with age. Previous studies have shown that age itself is a risk factor for LRIs, with children and the elderly at higher risk of severe complications due to a lack of specific and effective immune responses [35,36]. Period effects suggested that the risk of mortality, DALYs, and incidence had gradually decreased over time, likely due to socio-economic development, healthcare infrastructure improvements, and advancements in vaccine development. Overall, our study found that earlier birth cohorts exhibited higher LRIs mortality, DALYs, and incidence rates compared to later cohorts, reflecting longer or more prolonged exposure to risk factors in the earlier cohorts.

Despite the considerable impact of child underweight, household air pollution from solid fuels, and child stunting on the global burden of LRIs, these factors are often underemphasized in public health discussions. In particular, child underweight accounts for the highest proportion of all risk factors affecting LRIs DALYs in children under five, highlighting the importance of addressing child nutrition in global health strategies [37]. Furthermore, over three billion people worldwide lack access to clean energy and rely on solid fuels for cooking, leading to notable exposure to fine particulate matter [38,39]. Fine particulate matter contributes to LRIs by impairing mucociliary function and inducing excessive inflammation, thereby increasing susceptibility to infection and weakening the immune response [40]. Therefore, governments should prioritize accelerating the transition to clean cooking fuels and improving household air quality.

Among the 35–64 age group, smoking exerts the greatest impact on LRIs disease burden among all risk factors, while demonstrating considerable gender disparities in LRIs burden. Studies have confirmed that smoking exacerbates immune dysfunction, thereby increasing the risk of severe respiratory infections [41]. Some studies have indicated that smoking has a marked gender difference [42,43]. Over the past decade, progress in reducing smoking prevalence has stagnated in many countries [44], underscoring the need for stronger tobacco control policies. Our study also found that the burden of LRIs attributable to high temperatures has been increasing among individuals aged 15 and older in recent decades. Previous research has emphasized the negative impact of non-optimal temperature on LRIs [16]. Therefore, strengthening early warning systems for high temperatures and extreme weather is recommended, integrating meteorological data with public health information to identify potential LRIs health risks during periods of elevated temperatures.

Our analysis demonstrated strong negative correlations between the ASDR of LRIs and SDI, as well as between SDI and the ASDR of LRIs attributable to household air pollution from solid fuels and no access to handwashing facilities. These findings indicate that higher socio-economic development is associated with lower LRIs burden, while household air pollution from solid fuels and no access to handwashing facilities disproportionately affect lower-SDI regions, consistent with existing evidence [13,45]. Addressing these determinants through infrastructure improvements and policy interventions, such as promoting clean cookstoves and improving sanitation, is crucial to reducing the LRIs burden in these settings.

From 1990 to 2021, etiological analysis of LRIs indicated dominance of *Streptococcus pneumoniae* as the leading bacterial cause of LRIs mortality and DALYs, aligning with prior global burden estimates [4,25]. While ASMR and ASDR declined across pathogens, the sustained burden, particularly in low- and middle-income countries, reflects ongoing challenges in vaccination coverage and healthcare access [4,46]. The greatest reduction was seen in RSV-related infections, with AAPC of −5.38 for ASMR and −5.40 for ASDR (*p* < 0.05), likely due to advances in pediatric care, maternal immunization, and monoclonal antibody use (e.g., palivizumab) in high-risk groups. Nonetheless, RSV remains a major cause of morbidity in children under five, highlighting the need for expanded vaccine implementation, especially in low- and middle-income countries [46,47]. Although global coverage of pneumococcal conjugate vaccines and *Haemophilus influenzae* type b vaccines reached 65% and 77%, respectively, in 2023, with many vulnerable populations still lacking access [48,49]. The WHO Immunization Agenda 2030 highlights strategies such as targeting zero-dose children, building trust to reduce vaccine hesitancy, and improving vaccine access across all age groups to reduce pneumonia mortality in high-burden areas [50]. To maximize the global benefits of vaccination, equitable distribution is essential. Pharmaceutical companies, non-governmental organizations, and governments must work together to eliminate barriers to vaccine access in low-income countries [51].

In high-burden regions, the most effective strategies to combat LRIs are multifaceted and interrelated, including the following: (1) strengthening primary healthcare systems is central, as it enhances the capacity for prevention, diagnosis, and treatment of LRIs, particularly in resource-limited settings [52]; (2) expanding vaccine coverage is also critical, given persistent challenges with low immunization rates in low- and middle-income countries [53,54]; (3) indoor air pollution increases the risk of LRI. Promoting clean energy can reduce this risk [55]; (4) improving child nutrition enhances immune resilience and lowers susceptibility to LRIs [7]; (5) encouraging proper hygiene practices and behavioral changes remains essential, with community health workers playing a key role in delivering primary care services [56,57]; and (6) at the policy level, local governments should develop context-specific public health policies and allocate resources strategically to strengthen healthcare infrastructure and train medical personnel. In resource-constrained areas, the effectiveness of LRIs prevention and control is closely tied to local-level policy implementation and resource distribution [58,59].

The study provides a comprehensive analysis of the global burden of LRIs, detailing the disease burden across regions, genders, and age groups and offering valuable insights into global trends and major risk factors. However, there are several limitations, including reliance on existing mortality, DALYs, and incidence estimates, which may be affected by underreporting and data quality issues, especially in low-income countries. Additionally, while major risk factors were addressed, the complex interactions of environmental, socio-economic, and genetic factors require further research. In the age–period–cohort model analysis, the use of two-year average data to represent the period from 2020 to 2024, due to the lack of detailed data, may have resulted in the loss of some potentially valuable information. Lastly, due to the lack of subnational data in GBD, the study could only assess differences at national and regional levels but not subnational heterogeneity in disease burden (e.g., urban–rural or interprovincial variations). The data gap constitutes a limitation of our study, and future research should incorporate more granular data to refine the analysis.

## 5. Conclusions

In summary, the global ASIR, ASMR, and ASDR of LRIs have generally declined, with a marked reduction in burden during the COVID-19 pandemic. However, the disease burden remains high in sub-Saharan Africa and low-SDI regions. In addition, males bear a higher burden than females. Among risk factors, smoking has the greatest impact on individuals aged 35–64, while high temperatures increasingly affect those aged 15 and above. Moreover, the impact of household air pollution from solid fuels and no access to handwashing facilities is most severe in low-SDI regions. *Streptococcus pneumoniae* remains the leading pathogen, while RSV shows the most marked decline among pathogens. Therefore, effective LRIs control requires a comprehensive approach, including strengthening primary healthcare, expanding vaccine coverage, reducing indoor air pollution, improving child nutrition, promoting hygiene, and implementing context-specific public health policies.

## Data Availability

Public information is available online (http://ghdx.healthdata.org/gbd-results-tool (accessed on 26 August 2024)).

## Supplementary Materials

The following supporting information can be downloaded at: https://www.mdpi.com/article/10.3390/tropicalmed10070180/s1, Table S1: Number, ASR, AAPC of mortality, DALYs, and incidence of lower respiratory infections in 21 regions and SDI regions; Table S2: Number of deaths, DALYs, and incidence of lower respiratory infections by sex and across five SDI regions; Table S3: The ASDR, ASMR, and their AAPCs for lower respiratory infections of different etiologies; Figure S1: Time trends in the burden of lower respiratory infections by sex. (a) Death (1980–2021), (b) DALYs (1990–2021), and (c) incidence (1990–2021); Figure S2: Time trends in the burden of lower respiratory infections in five SDI regions. (a) Death (1980–2021); (b) DALYs (1990–2021), (c) incidence (1990–2021); Figure S3: Gender-specific burden of lower respiratory infections in 2021 and trends over time. (a) ASMR, ASDR, and ASIR in 2021; (b) AAPC of ASMR from 1980 to 2021, AAPC of ASDR, and ASIR from 1990 to 2021; Figure S4: The correlation between SDI and ASDR of lower respiratory infections attributable to risk factors.

## Abbreviations

The following abbreviations are used in this manuscript:

LRIs	Lower respiratory infections
DALYs	Disability-adjusted life years
SDI	Socio-demographic index
ASMR	Age-standardized mortality rate
ASDR	Age-standardized disability-adjusted life years rate
ASIR	Age-standardized incidence rate
APC	Age–period–cohort
AAPC	Average annual percentage change
CI	Confidence intervals
UI	Uncertainty interval
RSV	Respiratory syncytial virus
