# Supplementary material for: Global Trends and Attributable Risk Factors in the Disease Burden of Lower Respiratory Infections
Source: Trop Med Infect Dis. 2025 Jun 26;10(7):180. doi: 10.3390/tropicalmed10070180 (PMC12300696; doi:10.3390/tropicalmed10070180)
Supplement: Supplementary file 1 [file tropicalmed-10-00180-s001.zip › tropicalmed-3637116-supplementary.pdf]

Global trends and attributable risk factors of disease burden of lower respiratory infections

E Yu[1]; Chunhui Li[1,*]

[1]Institute of Data Science and Big Data Technology, School of Mathematics and Physics, Wuhan Institute of Technology, Wuhan, Hubei, People's Republic of China. Yu: 22310010026@stu.wit.edu.cn

*Corresponding author: Chunhui Li, Institute of Data Science and Big Data Technology, School of Mathematics and Physics, Wuhan Institute of Technology, Wuhan, Hubei, People's Republic of China. E-mail: chli0201@wit.edu.cn

Captions

Supplementary Table S1. Number, ASR, and AAPC of mortality, DALYs and incidence of lower respiratory infections in 21 regions and SDI regions.

Supplementary Table S2. Number of deaths, DALYs, and incidence of lower respiratory infections by sex and across five SDI regions.

Supplementary Table S3. The ASDR, ASMR and their AAPCs for lower respiratory infections of different etiologies.

Supplementary Figure. S1. Time trends in the burden of lower respiratory infections by sex. (a) Death (1980-2021), (b) DALYs (1990-2021), (c) incidence (1990-2021).

Supplementary Figure. S2. Time trends in the burden of lower respiratory infections in five SDI regions. (a) Death (1980-2021); (b) DALYs (1990-2021), (c) incidence (1990-2021).

Supplementary Figure. S3. Gender-specific burden of lower respiratory infections in 2021 and trends over time. (a) ASMR, ASDR, and ASIR in 2021; (b) AAPC of ASMR from 1980 to 2021, AAPC of ASDR, and ASIR from 1990 to 2021.

Supplementary Figure. S4. The correlation between SDI and ASDR of lower respiratory infections attributable to risk factors.

Supplementary Table S1. Number, ASR, and AAPC of mortality, DALYs and incidence of lower respiratory infections in 21 regions and SDI regions.

location	1980		1990				2021						Average annual percentage change		
	Death Number	ASMR	DALYs	ASDR	Incidence Number	ASIR	Death Number	ASMR	DALYs	ASDR	Incidence Number	ASIR	ASMR (1980-2021)	ASDR (1990-2021)	ASIR (1990-2021)
Global	3424140 (3059020, 3882329)	78(70.7, 86.3)	204174056(180683528, 229006748)	3472.9(3090.7, 3872.1)	313864642 (294055265, 333297862)	6373.2(59 93.5, 6746)	2183001 (1979915, 2360084)	28.7(26, 31.1)	82534840(72611990, 93402507)	1168.8(1017, 1336.9)	343606787 (325214314, 363517285)	4283.6(4057, 4524.9)	-2.4*(-2.6,-2.3)	-3.5*(-3.6,-3.4)	-1.3*(-1.4,-1.2)
Low SDI	782720 (647786, 949826)	164.4(144 , 187.1)	58995960(48385985, 70445378)	7438.1(6344.8, 8574.9)	54637166 (50881132, 58157068)	13580.1(12769.8, 14376)	502759 (429167, 581033)	70.7(62.6, 78.6)	30675991(24877530, 36959732)	2580.6(2198.7, 2993.4)	68642108 (64950787, 72639931)	9261.1(8741. 6, 9820.9)	-2.1*(-2.2,-2.0)	-3.4*(-3.6,-3.3)	-1.3*(-1.4,-1.2)
Low-middle SDI	1184085 (1034465, 1364918)	105.2(93. 9, 117.9)	72425785(63526550, 81680170)	4608.4(4088.8, 5139.4)	119090743 (111451635, 127070318)	12914.6(12086.1, 13665.2)	594109 (525288, 658730)	43.1(38.1, 47.7)	26753151(23181483, 30505072)	1575.1(1374.9, 1781.3)	129529835(122092996, 137811377)	8258.5(7738. 3, 8773.6)	-2.2*(-2.5,-2.0)	-3.5*(-3.6,-3.3)	-1.5*(-1.6,-1.3)
Middle SDI	900864 (822405, 992965)	75.5(69, 82)	53988568(49096688, 59431802)	2994.7(2744.8, 3259.2)	87083350 (81429776, 92867529)	6136.4(5786.1, 6472.5)	542885 (493805, 588398)	25.4(23, 27.6)	15753940(14373223, 17131133)	762.1(692.3, 834.2)	89548178(84651289, 95296608)	3682.8(3486. 7, 3914.4)	-2.6*(-2.8,-2.5)	-4.4*(-4.5,-4.2)	-1.7*(-1.8,-1.6)
High-middle SDI	316014 (292177, 344410)	42.2(39,4 5.5)	13601574(12383745, 15168448)	1499.5(1365.4, 1674.6)	37205343 (34882780, 39679471)	3875.9(3635.7, 4122.2)	241824 (215370, 266650)	13.8(12.4, 15.2)	4955001(4612544, 5340711)	341(318, 367.6)	40930228(38490019, 43545701)	2707.6(2540. 9, 2896.8)	-2.7*(-3.0,-2.4)	-4.7*(-5.0,-4.4)	-1.2*(-1.3,-1.1)
High SDI	238115 (222923, 246229)	29.4(27.2, 30.6)	5031269(4738376, 5206311)	525.4(496, 546.1)	15687702 (14862527, 16622907)	1661.1(1574.3, 1765.3)	299446 (253666, 324184)	12.1(10.4, 13)	4325461(3856390, 4586605)	217.1(198.7, 227.9)	14820503(14061955, 15679451)	891.1(844.6, 942.6)	-2.1*(-2.7,-1.6)	-2.8*(-3.3,-2.2)	-2.0*(-2.1,-1.9)

Central Asia	66874(61010,71866)	93.5(86,99.9)	4569914(4274053,4890479)	4997.6(4687.4,5338.1)	2603120(2459125,2768856)	3569.6(3396.5,3762.6)	19996(17590,22430)	23.3(20.7,26)	1706273(1618078,1803193)	1280.9(1105.3,1488.8)	1706273(1618078,1803193)	1958.1(1855.2,2064.7)	-3.3*(-3.7,-2.9)	-4.1*(-4.5,-3.8)	-1.8*(-2,-1.7)
Central Europe	39747(38444,41341)	36.1(34.7,37.6)	1190128(1150335,1231424)	1159.5(1114.7,1205.3)	2317023(2191783,2456731)	1969.6(1860.6,2103)	33047(30004,35141)	15.5(14.2,16.5)	1274221(1208629,1344161)	399.5(370.7,427)	1274221(1208629,1344161)	888.4(839,941.8)	-2.0*(-2.7,-1.2)	-3.3*(-3.9,-2.6)	-2.5*(-2.6,-2.4)
Eastern Europe	44744(43553,45960)	23.6(22.9,24.4)	1310729(1275854,1346463)	729.2(706.6,751.8)	5898623(5516256,6284876)	2634.8(2445.9,2828.8)	29780(27272,32607)	10.3(9.4,11.2)	3972021(3747231,4202394)	400.3(370.1,433.4)	3972021(3747231,4202394)	1526.6(1429.5,1624.4)	-2.1*(-3.3,-1.0)	-1.7*(-3.0,-0.4)	-1.7*(-1.8,-1.6)
Australasia	2886(2634,3088)	19.3(17.4,20.7)	52475(49369,55341)	261.9(246.1,276.2)	210296(194608,226821)	994.9(927.2,1070.6)	4335(3539,4804)	6.7(5.5,7.4)	228074(212298,243210)	100.7(88,108.9)	228074(212298,243210)	497.4(466,530.2)	-2.6*(-3.8,-1.4)	-3.1*(-4.7,-1.4)	-2.2*(-2.5,-1.9)
High-income Asia Pacific	42872(39892,44673)	39.4(35.8,41.4)	1194416(1109815,1241461)	704.6(650,737.1)	2250118(2115850,2402725)	1502(1401.6,1620.1)	96858(78185,107835)	14.3(12,15.7)	1755279(1663982,1858055)	219.9(194.4,235.1)	1755279(1663982,1858055)	636.2(588.4,687.5)	-2.3*(-2.7,-2.0)	-3.6*(-4.1,-3.1)	-2.7*(-2.8,-2.6)
High-income North America	53551(48763,56255)	19.2(17.3,20.2)	1415968(1318251,1473261)	427.2(400.1,442.9)	6348162(5991596,6738520)	1932.9(1831,2049.2)	60868(51676,65939)	8.8(7.6,9.5)	5642677(5314602,6009291)	183.7(170.1,194.8)	5642677(5314602,6009291)	1003.3(949.9,1061.5)	-1.8*(-2.8,-0.9)	-2.6*(-3.5,-1.7)	-2.1*(-2.1,-2)
Southern Latin America	13619(13043,14149)	37.9(36,39.4)	462599(447572,476376)	986.7(953.9,1016.6)	1091415(1022643,1167974)	2316.1(2177.8,2467.4)	35588(31486,38256)	39.4(35,42.3)	1216383(1142429,1292514)	738.2(680.6,787.1)	1216383(1142429,1292514)	1563.4(1474,1660.8)	0.1(-0.5,0.8)	-0.9(-2.0,0.2)	-1.4*(-1.6,-1.2)
Western Europe	135848(126778,140953)	30.7(28.4,32)	1812536(1694626,1875546)	357(336.2,368.2)	4393220(4155184,4658309)	903.7(855.7,961.7)	123133(102349,133809)	10(8.5,10.8)	4133339(3885562,4394574)	155(138.3,163.9)	4133339(3885562,4394574)	524.6(493.9,556.7)	-2.8*(-3.8,-1.7)	-2.6*(-3.7,-1.4)	-1.7*(-1.8,-1.7)
Andean Latin America	48150(42486,54386)	170.5(153.7,186.2)	2110168(1871668,2379984)	4685(4234.7,5194.5)	2225745(2065744,2395520)	6758.6(6353.3,7177.8)	29552(23960,36139)	52.2(42.3,63.8)	1814627(1663373,1947357)	1221.9(1004.5,1485.3)	1814627(1663373,1947357)	3021.3(2764.6,3245.6)	-3.0*(-3.7,-2.3)	-4.5*(-5.2,-3.8)	-2.7*(-3.0,-2.5)

Caribbean	17936(15342,20861)	72.2(64.8,80)	962324(830130,1109605)	2519.1(2199.7,2866.4)	1299575(1217036,1387074)	4098(3868.1,4348.7)	16708(14754,18893)	32.8(28.6,37.1)	1011577(955217,1072679)	1196.2(956.6,1461.5)	1011577(955217,1072679)	2061.6(1950.3,2190.4)	-1.9*(-2.3,-1.5)	-2.3*(-2.7,-2.0)	-2.2*(-2.3,-2.1)
Central Latin America	91160(86290,96330)	83.5(79.7,86.8)	3908056(3680254,4179155)	2043.8(1942.7,2163.6)	4481838(4189106,4849695)	3104(2936.8,3286.9)	51353(45642,57423)	21.9(19.5,24.6)	2929885(2778053,3103210)	671.3(581.6,784.8)	2929885(2778053,3103210)	1232.8(1165.7,1308.5)	-3.2*(-3.7,-2.7)	-3.5*(-4.1,-2.9)	-2.9*(-3.0,-2.9)
Tropical Latin America	72745(65831,81444)	63.9(59.2,69)	3159472(2908781,3449279)	2232.5(2074.1,2409.5)	7805613(7345341,8366672)	6361.2(6027.3,6722.7)	79308(69382,86053)	32.8(28.6,35.6)	7110826(6655708,7649804)	723.3(657.7,782.7)	7110826(6655708,7649804)	3069.2(2865.7,3305.2)	-1.6*(-2.3,-0.9)	-3.6*(-4.0,-3.1)	-2.3*(-2.9,-1.8)
North Africa and Middle East	302863(260298,382279)	83.6(74,99.4)	13938547(12052555,17286606)	3039.2(2659.9,3678.2)	11152161(10188124,12205567)	3525.6(3290.8,3751.9)	92168(81359,104416)	22.7(19.9,25.6)	9378540(8816051,9978197)	709.1(616,810.7)	9378540(8816051,9978197)	1857(1756.2,1968.6)	-3.1*(-3.3,-3.0)	-4.6*(-4.7,-4.5)	-2.0*(-2.1,-2.0)
South Asia	1001946(853597,1176678)	97.1(84.4,109.9)	61399105(52073669,69735872)	4317.8(3717,4854)	143230712(134148003,152783427)	17288.5(16165.5,18335)	515691(452545,584513)	39.2(34.4,44.9)	172090100(161275839,184376381)	1507(1299.1,1732.2)	172090100(161275839,184376381)	11101.2(10358,11891.2)	-2.2*(-2.7,-1.8)	-3.5*(-3.7,-3.2)	-1.5*(-1.7,-1.3)
East Asia	502027(443185,571953)	74(66.1,82.8)	33104913(28636621,38032656)	3067.7(2674.2,3502.7)	52834282(49329072,56586893)	5487.5(5158,5841.4)	224461(187516,269818)	14.5(12.2,17.4)	46575109(43573065,49747290)	355.3(307.9,411.1)	46575109(43573065,49747290)	2861(2675.6,3073.6)	-4.0*(-4.2,-3.7)	-6.9*(-7.2,-6.6)	-2.1*(-2.2,-2.0)
Oceania	5501(4176,6692)	105(84.7,123.1)	449910(367147,553967)	5021.8(4234.7,5987.1)	373633(345704,404840)	6784(6353.9,7235.6)	6710(5484,8196)	56.8(47.5,70.3)	460061(427101,492542)	2823.2(2282,3449.9)	460061(427101,492542)	4183.7(3907.4,4441.8)	-1.5*(-1.6,-1.4)	-1.9*(-2.2,-1.6)	-1.5*(-1.6,-1.5)
Southeast Asia	306135(262697,364785)	76.8(67.3,89.6)	17361960(15281716,20310517)	3315.8(2952.6,3811.9)	21438667(19858617,23076343)	5265.9(4924.6,5596)	200357(172210,221536)	38.4(32.7,42.6)	20271944(19086778,21509075)	1079.4(944,1215.3)	20271944(19086778,21509075)	3291.9(3102.3,3486)	-1.7*(-1.8,-1.6)	-3.6*(-3.7,-3.5)	-1.5*(-1.6,-1.4)
Central Sub-Saharan Africa	70366(55852,85377)	184.9(151.4,228.1)	5900147(4459005,7271046)	7269.9(5839.2,8711.1)	4581762(4242016,4938602)	10590.8(9899.5,11313.7)	61639(49195,77657)	106.7(81.8,138.6)	6028644(5555553,6487018)	2860.4(2268.2,3619.3)	6028644(5555553,6487018)	7044.6(6553,7611.5)	-1.3*(-1.4,-1.3)	-3.0*(-3.1,-2.9)	-1.3*(-1.4,-1.2)

Eastern Sub-Saharan Africa	262704(217331,317333)	179.3(155.1,202.2)	21613674(17754087,26326885)	7551(6530.5,8817.6)	19607674(18283182,20927589)	12929.4(12197.7,13612.8)	172757(150812,195778)	81.9(72.4,90.6)	24559928(23113417,26016128)	2471.7(2159.6,2795.5)	24559928(23113417,26016128)	9102.5(8589.4,9658.4)	-1.9*(-2.0,-1.9)	-3.6*(-3.8,-3.4)	-1.2*(-1.4,-0.9)
Southern Sub-Saharan Africa	45936(41012,51276)	126.6(114.4,141.1)	2506284(2251513,2824717)	4298.7(3897.5,4732.7)	4344790(4084525,4601286)	9760.3(9207.2,10279.7)	44082(39086,49141)	76.3(67.9,84.3)	5724742(5371919,6066004)	2664.8(2337,2997.2)	5724742(5371919,6066004)	8469.4(7959,9004.2)	-1.3*(-1.6,-1.1)	-1.6*(-2,-1.2)	-0.6*(-0.8,-0.3)
Western Sub-Saharan Africa	296532(242641,357723)	163.9(139.5,188)	25750730(20609158,30839736)	8126.6(6791.4,9537.9)	15376215(14326898,16429869)	9131.4(8575.3,9643.7)	284611(221050,354111)	85.5(71,99.8)	25722537(24224441,27255902)	3258.7(2537,4045.2)	25722537(24224441,27255902)	8000.2(7571.2,8447.2)	-1.6*(-1.7,-1.5)	-2.9*(-3.1,-2.8)	-0.4*(-0.5,-0.4)

Notes: * $p < 0.05$, ASR, age-standardized rate; DALYs, disability-adjusted life years; SDI, socio-demographic index; AAPC, average annual percentage change.

Supplementary Table S2. Number of deaths, DALYs, and incidence of lower respiratory infections by sex and across five SDI regions.

	1980	1990		2019			2021			Percentage change			Percentage change		
	Death	DALYs	Incidence	Death	DALYs	Incidence	Death	DALYs	Incidence	Death (1980-2019)	DALYs (1990-2019)	Incidence (1990-2019)	Death (2019-2021)	DALYs (2019-2021)	Incidence (2019-2021)
Sex															
Both	3424140.25(3059020.41,3882329.25)	204174056.79(180683528.72,229006748.29)	313864642.09(294055264.90,333297862.17)	2549851.71(2316876.34,2749435.73)	103323270.98(91618467.27,116062311.05)	369174683.31(349123696.83,390500071.71)	2183001.29(1979915.46,2360083.92)	82534840.54(72611990.16,93402507.14)	343606787.39(325214314.01,363517284.75)	-25.53(-35.70,-13.77)	-49.39*(-49.29,-49.32)	17.62*(18.73,17.16)	-14.39*(-14.54,-14.16)	-20.12*(-20.75,-19.52)	-6.93*(-6.85,-6.91)
Male	1753581.36(1557458.55,2008205.83)	106820686.95(93919781.74,121095966.10)	166991424.43(156457643.57,177508398.28)	1355042.38(1252875.70,1466594.53)	55826732.01(49084733.63,62873471.04)	200173467.05(189062809.21,211964305.07)	1172230.26(1078492.90,1269919.54)	45402003.66(39793008.47,51982462.64)	183254768.49(173853205.48,194137697.71)	-22.73*(-33.46,-10.27)	-47.74*(-47.74,-48.08)	19.87*(20.84,19.41)	-13.49*(-13.92,-13.41)	-18.67*(-18.93,-17.32)	-8.45*(-8.04,-8.41)
Female	1670558.89(1465829.93,1911694.78)	97353369.84(84903574.42,110194838.17)	146873217.65(137447465.80,156213059.29)	1194809.32(1047153.02,1304139.93)	47496538.98(41113361.94,53127569.03)	169001216.26(159881028.14,178810875.62)	1010771.03(878596.25,1128326.87)	37132836.88(31972682.19,41568754.28)	160352018.90(151438077.82,169599996.71)	-28.48*(-39.80,-15.03)	-51.21*(-51.58,-51.79)	15.07*(16.32,14.47)	-15.40*(-16.10,-13.48)	-21.82*(-22.23,-21.76)	-5.12*(-5.28,-5.15)
SDI															
High SDI	238114.83(222923.05,246229.80)	5031268.85(4738376.34,5206310.67)	15687702.45(14862526.51,16622906.55)	363051.24(310027.27,392072.83)	5293972.85(4749639.11,5605206.60)	17908405.94(16984955.86,18902469.10)	299446.00(253666.31,324184.21)	4325460.96(3856389.82,4586605.37)	14820503.33(14061954.51,15679451.40)	52.47*(34.22,73.20)	5.22*(0.24,7.66)	14.16*(14.28,13.71)	-17.52*(-18.18,-17.32)	-18.29*(-18.81,-18.17)	-17.24*(-17.21,-17.05)
High-middle SDI	316014.23(292176.71,344410.35)	13601573.99(12383745.23,15168448.37)	37205342.89(34882779.54,39679471.48)	275441.35(248883.75,295947.52)	5836153.11(5504651.92,6163788.21)	45852931.47(43017341.32,48937999.50)	241824.11(215370.04,266650.02)	4955001.26(4612544.40,5340710.64)	40930228.45(38490018.56,43545700.66)	-12.84*(-22.65,-1.78)	-57.09*(-55.55,-59.36)	23.24*(23.32,23.33)	-12.20*(-13.47,-9.90)	-15.10*(-16.21,-13.35)	-10.74*(-10.52,-11.02)
Middle SDI	900864.46(822405.10,992965.18)	53988568.35(49096688.33,59431802.52)	87083350.15(81429775.75,92867528.61)	605450.45(552741.77,646047.62)	19273733.54(17606798.25,20931579.20)	96729301.91(91093087.67,102498829.13)	542885.31(493805.26,588397.65)	15753940.05(14373223.25,17131132.75)	89548177.98(84651288.53,95296608.34)	-32.79*(-40.53,-24.05)	-64.30*(-64.14,-64.78)	11.08*(11.87,10.37)	-10.33*(-10.66,-8.92)	-18.26*(-18.37,-18.16)	-7.42*(-7.07,-7.03)
Low-middle SDI	1184085.38(1034465.64,1364918.55)	72425784.76(63526550.03,81680170.13)	119090743.25(111451634.87,127070318.13)	711947.64(639328.53,778886.53)	35072731.63(30845105.26,39843625.51)	137058929.12(129327437.53,145455615.24)	594109.14(525288.03,658729.59)	26753150.61(23181483.17,30505072.31)	129529834.86(122092995.91,137811376.96)	-39.87*(-49.28,-28.72)	-51.57*(-51.45,-51.22)	15.09*(16.04,14.47)	-16.55*(-17.84,-15.43)	-23.72*(-24.85,-23.44)	-5.49*(-5.59,-5.26)
Low SDI	782720.20(647786.88,949826.01)	58995959.83(48385985.40,70445378.11)	54637166.52(50881131.75,58157068.09)	591591.53(510787.07,678801.58)	37757875.09(31189134.75,44924436.29)	71468320.49(67306266.64,75430080.05)	502758.56(429166.67,581033.94)	30675991.51(24877529.87,36959731.98)	68642108.62(64950786.72,72639931.09)	-24.42*(-40.45,-4.07)	-36.00*(-35.54,-36.23)	30.81*(32.28,29.70)	-15.02*(-15.98,-14.40)	-18.76*(-20.24,-17.73)	-3.95*(-3.50,-3.70)

Notes:[*] $p < 0.05$. DALYs, disability-adjusted life years; SDI, socio-demographic index.

Supplementary Table S3. The ASDR, ASMR and their AAPCs for lower respiratory infections of different etiologies.

Etiologies	1990		2021		1990-2021	
	ASMR	ASDR	ASMR	ASDR	AAPC for Deaths (95% CI)	AAPC for DALYs (95% CI)
Influenza	5.87(5.33,6.40)	304.31(267.40,346.58)	1.30(0.98,1.66)	59.69(44.18,77.17)	-4.96*(-5.31,-4.60)	-5.61*(-6.01,-5.20)
Group B streptococcus	1.25(1.10,1.42)	78.46(65.70,91.64)	0.74(0.65,0.83)	35.89(30.31,42.19)	-1.68*(-1.84,-1.52)	-2.49*(-2.58,-2.41)
Acinetobacter baumannii	2.30(1.93,2.78)	104.18(78.87,137.36)	0.97(0.83,1.14)	37.84(29.01,50.39)	-2.75*(-2.88,-2.62)	-3.22*(-3.31,-3.14)
Respiratory syncytial virus	2.31(2.05,2.60)	193.49(170.61,219.46)	0.49(0.36,0.65)	40.82(29.91,54.59)	-5.38*(-5.84,-4.92)	-5.40*(-5.82,-4.97)
Enterobacter spp	0.51(0.42,0.65)	25.98(18.59,37.60)	0.39(0.33,0.45)	14.33(11.17,18.89)	-0.87*(-1.02,-0.72)	-1.90*(-1.99,-1.81)
Fungus	0.75(0.63,0.87)	25.86(20.96,31.29)	0.56(0.48,0.64)	16.37(13.89,19.15)	-0.91*(-1.05,-0.77)	-1.46*(-1.58,-1.35)
Escherichia coli	2.04(1.79,2.30)	105.58(88.92,125.24)	1.32(1.16,1.48)	51.98(43.41,61.42)	-1.38*(-1.57,-1.18)	-2.27*(-2.40,-2.14)
Haemophilus influenzae	2.51(2.20,2.87)	165.74(139.24,196.74)	0.76(0.68,0.86)	37.50(31.03,43.96)	-3.76*(-3.96,-3.55)	-4.72*(-4.86,-4.58)
Mycoplasma	1.46(1.31,1.64)	88.33(76.99,103.09)	0.78(0.69,0.88)	41.01(34.44,47.85)	-1.96*(-2.10,-1.83)	-2.46*(-2.63,-2.29)
Staphylococcus aureus	6.11(5.54,6.56)	242.89(216.69,270.69)	5.43(4.89,5.90)	156.80(139.44,176.08)	-0.36*(-0.56,-0.15)	-1.40*(-1.57,-1.24)
Chlamydia spp	1.80(1.59,2.00)	107.67(91.77,123.24)	0.73(0.64,0.81)	36.29(30.66,42.36)	-2.89*(-2.99,-2.80)	-3.45*(-3.53,-3.37)
Legionella spp	1.06(0.92,1.22)	37.85(29.09,48.79)	0.86(0.76,0.95)	24.74(20.88,29.35)	-0.63*(-1.00,-0.25)	-1.35*(-1.45,-1.24)
Other viral etiologies of LRI	3.41(3.09,3.77)	228.69(202.42,259.47)	1.65(1.47,1.83)	83.79(71.21,96.47)	-2.30*(-2.42,-2.17)	-3.20*(-3.29,-3.10)
Other bacterial and viral pathogens	3.05(2.69,3.37)	101.46(87.33,116.64)	1.77(1.54,1.97)	48.33(41.68,55.24)	-1.74*(-1.97,-1.50)	-2.36*(-2.45,-2.27)
Pseudomonas aeruginosa	2.01(1.82,2.19)	93.49(82.53,106.24)	1.61(1.44,1.75)	56.79(49.40,64.82)	-0.68*(-0.88,-0.48)	-1.59*(-1.77,-1.42)
Streptococcus pneumoniae	20.01(18.11,21.99)	1249.50(1097.12,1415.86)	6.69(5.98,7.35)	309.90(264.44,357.22)	-3.47*(-3.62,-3.32)	-4.41*(-4.47,-4.35)
Klebsiella pneumoniae	4.79(4.31,5.27)	281.51(242.56,321.31)	2.32(2.09,2.58)	99.46(84.65,115.82)	-2.29*(-2.37,-2.20)	-3.31*(-3.41,-3.20)
Polymicrobial	0.54(0.37,0.75)	36.31(22.26,56.69)	0.28(0.19,0.39)	16.33(9.85,25.88)	-2.09*(-2.17,-2.02)	-2.54*(-2.62,-2.46)

Notes: * $p < 0.05$. ASMR, age-standardized mortality rate; ASDR, age-standardized disability-adjusted life years rate; AAPC, Average annual percentage change.

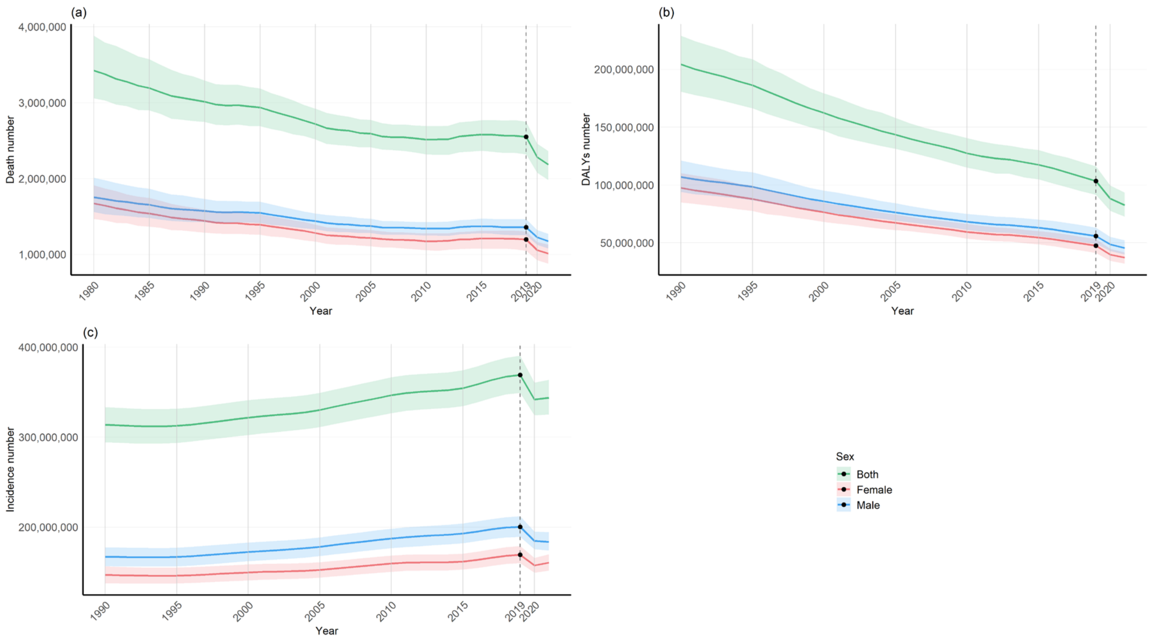

Supplementary Figure. S1. Time trends in the burden of lower respiratory infections by sex. (a) Death (1980-2021), (b) DALYs (1990-2021), (c) incidence (1990-2021). DALYs, disability-adjusted life years.

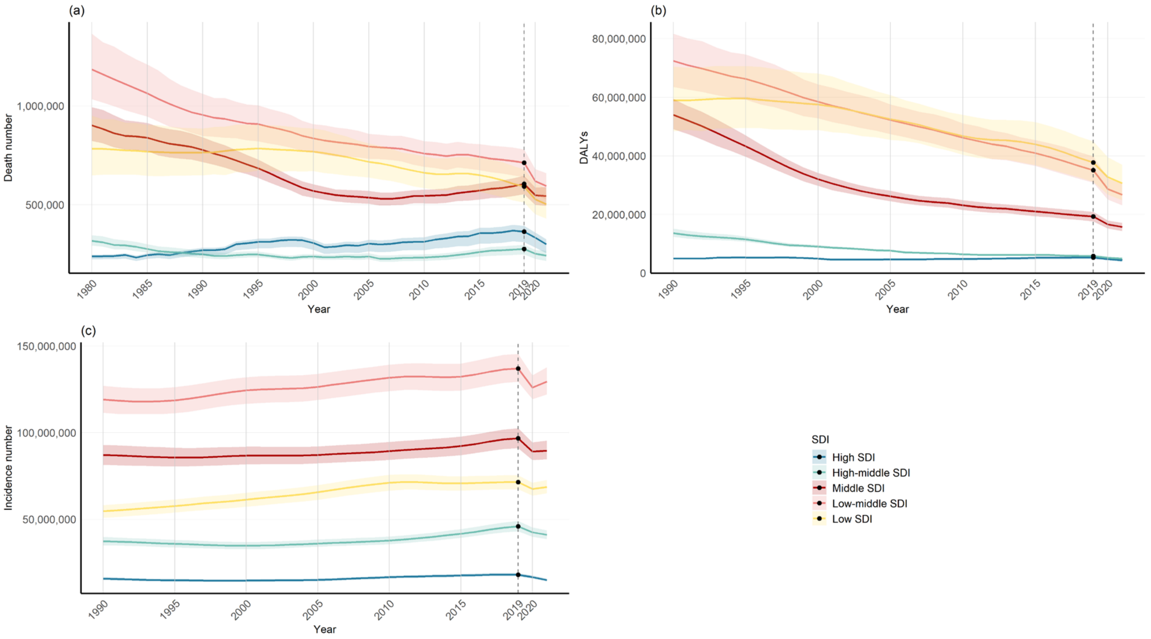

Supplementary Figure. S2. Time trends in the burden of lower respiratory infections in five SDI regions. (a) Death (1980-2021); (b) DALYs (1990-2021), (c) incidence (1990-2021). SDI, socio-demographic index; DALYs, disability-adjusted life years.

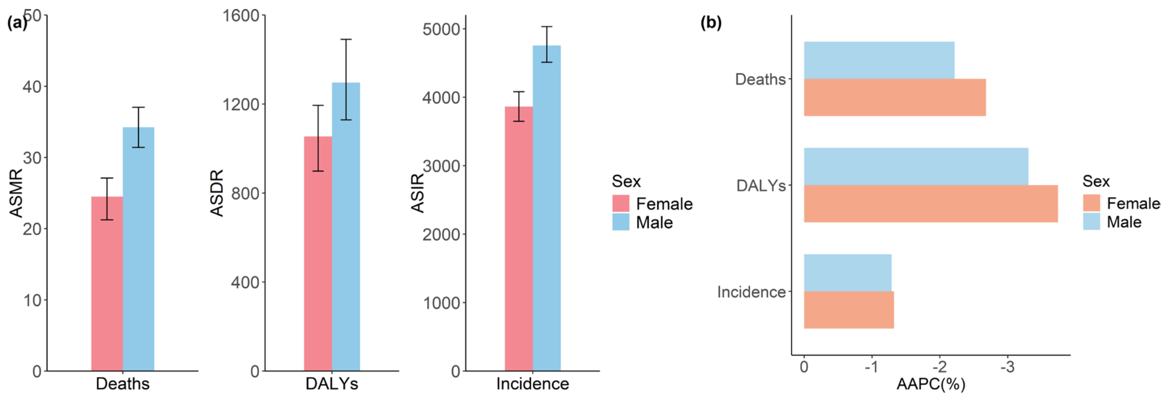

Supplementary Figure. S3. Gender-specific burden of lower respiratory infections in 2021 and trends over time. (a) ASMR, ASDR, and ASIR in 2021; (b) AAPC of ASMR from 1980 to 2021, AAPC of ASDR, and ASIR from 1990 to 2021. ASMR, age-standardized mortality rate; ASDR, age-standardized disability-adjusted life years rate; ASIR, age-standardized incidence rate; AAPC, average annual percentage change; DALYs, disability-adjusted life years.

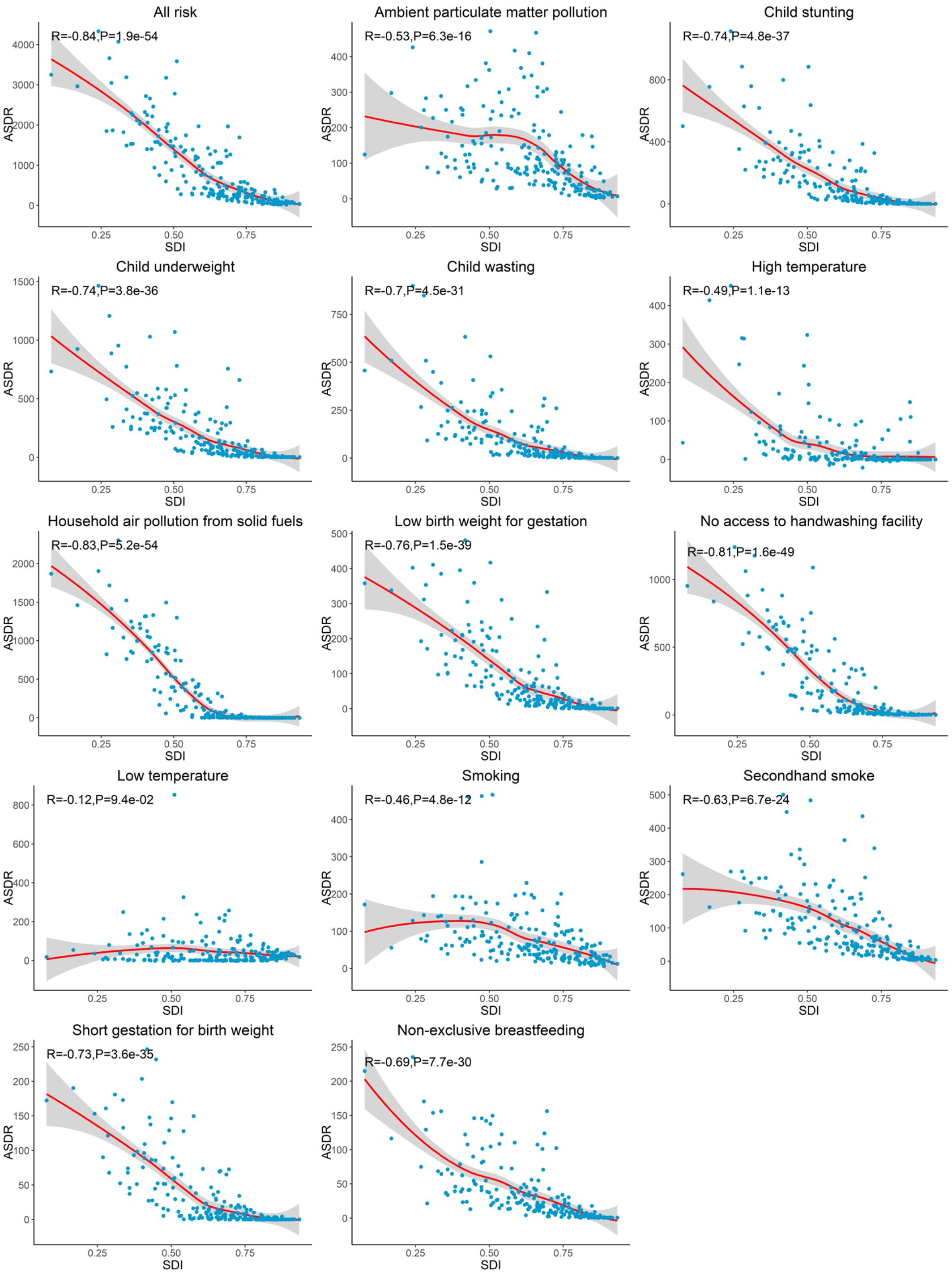

Supplementary Figure. S4. The correlation between SDI and ASDR of lower respiratory infections attributable to risk factors. SDI, socio-demographic index; ASDR, age-standardized disability-adjusted life years rate.
